# RETENTION OF DUALLY ELIGIBLE BENEFICIARIES IN ASSISTED LIVING AT THE END OF LIFE

**DOI:** 10.1093/geroni/igac059.1656

**Published:** 2022-12-20

**Authors:** Susan Hayes, Nicole Rosendaal, Xiao (Joyce) Wang, Kali Thomas, Emma Belanger

**Affiliations:** Brown University School of Public Health, Providence, Rhode Island, United States; Brown University, Providence, Rhode Island, United States; Brown University, Providence, Rhode Island, United States; Brown University, Providence, Rhode Island, United States; Brown University, Providence, Rhode Island, United States

## Abstract

To examine to what extent dually eligible beneficiaries (duals) residing in assisted living remain there toward the end of life, we conducted a prospective cohort study of 98,944 Medicare beneficiaries present at validated AL ZIP codes in January 2017, and who died during a two-year follow-up. The outcome was AL residence in the last 30 days of life. We compared decedents who were not duals (80,156 decedents), with those newly dually eligible in 2017-2018 (3,722 decedents), and those already dually eligible in 2016 (15,066 decedents). Only 36.7% of new dual decedents resided in AL in the last 30 days of life, compared to 66.2% among those dually eligible in 2016, and 84.5% of those without Medicaid. While 29 states retained over half of all decedents in AL until death, only 8 states retained a majority of dually eligible decedents.

